# The Validity of a Smartphone App to Serve as Proxy for Exposure Time and Activity Level in Functionally Active Patients With Knee Osteoarthritis and Young Athletes Post-ACL Reconstruction

**DOI:** 10.1177/23259671261466479

**Published:** 2026-09-23

**Authors:** Nicole K. Bryant, Alan Getgood, Jinhui Ma, Kevin R. Willits, Robert Litchfield, J. Robert Giffin, Ryan M. Degen, Trevor B. Birmingham, Dianne M. Bryant

**Affiliations:** * Fowler Kennedy Sport Medicine Clinic, London, Ontario, Canada; † Department of Epidemiology and Biostatistics, Schulich School of Medicine and Dentistry, Western University, London, Ontario, Canada; ‡ Western University Bone and Joint Institute, London, Ontario, Canada; § Western University Collaborative Training Specialization in Musculoskeletal Health Research, London, Ontario, Canada; ‖ Department of Surgery, Schulich School of Medicine and Dentistry, Western University, London, Ontario, Canada; ¶ Fortius Clinic London, London, United Kingdom; # Department of Health Research Methods, Evidence, and Impact, McMaster University, Hamilton, Ontario, Canada; ** School of Physical Therapy, Faculty of Health Sciences, Western University, London, Ontario, Canada; Investigation performed at Fowler Kennedy Sports Medicine Clinic, 3M Centre, London, Ontario, Canada

**Keywords:** anterior cruciate ligament reconstruction, Bland-Altman agreement, exposure time, knee osteoarthritis, Marx Activity Rating Scale, smartphone activity tracking

## Abstract

**Background:**

Accurate measurement of physical activity and exposure time is essential for evaluating risk, prevention, and treatment strategies, but traditional accelerometry and manual tracking are costly and burdensome. Smartphone step counting offers a scalable alternative, yet its validity in orthopaedic populations is uncertain.

**Purpose:**

To test whether smartphone step count provides a valid proxy for exposure time and activity level in functionally active patients with knee osteoarthritis (OA), and young athletes post–anterior cruciate ligament reconstruction (ACLR).

**Study Design:**

Cohort study (Diagnosis); Level of evidence, 2.

**Methods:**

Participants tracked activity for 4 weeks using a smartphone app and detailed activity diaries; secondary measures included the Marx Activity Rating Scale (MARS) and Sport Participation questionnaire. Correlations were assessed using Spearman's ρ, agreement using Bland-Altman analysis, and predictability using rank regression.

**Results:**

A total of 102 participants contributed data (ACLR: n = 47; 51% female; 24 ± 4 years; OA: n = 55; 44% female; 58 ± 11 years). The OA and ACLR groups showed poor correlations between app step count and diary-reported activity (OA: ρ = 0.17; *P* = .20; ACLR: ρ = 0.27; *P* = .07). Bland-Altman plots demonstrated minimal systematic bias with mean differences near zero, but wide limits of agreement (LoA) indicating poor interchangeability for individual monitoring (OA: d̄ = −0.10, LoA [–2.49 to 2.29]; ACLR: d̄ = −0.17, LoA [–1.93 to 2.28]). MARS correlated moderately with diary-reported activity (OA: ρ = 0.33; *P* = .02; ACLR: ρ = 0.45; *P* = .002), with stronger associations in ACLR participants. Sport participation was moderately correlated with diary-reported activity in the ACLR group. MARS scores were significant predictors of diary-reported activity in both groups, and sport participation was a significant predictor in the ACLR group. Step counts did not predict diary-reported activity.

**Conclusion:**

Our study demonstrated acceptable group-level agreement between the smartphone app and diary-based activity but insufficient precision for individual patient monitoring. MARS outperformed an app-based step count as a proxy for exposure time, particularly in athletes.

Athletes recovering from injury often aim to return to sport as quickly as possible.^14^ However, returning to activity increases an athlete's time exposed to the elevated risk of reinjury (exposure time).^2,4,15,20,22^ Previous injury is among the strongest predictors of future injury, and athletes returning to sport are significantly more likely to experience subsequent injuries than their uninjured counterparts.^27,30^ Therefore, accurately quantifying exposure time is essential for evaluating the effectiveness of injury-prevention or rehabilitation strategies.^9^ In this context, exposure time refers to the amount of time an individual participates in sport or other at-risk physical activity.^29^ For example, in a study comparing 2 anterior cruciate ligament reconstruction (ACLR) techniques, if 1 approach delays readiness for return to sport (eg, causes slower recovery of strength or donor site morbidity that affects the amount of pain for longer, etc), comparing graft failure rates between groups at a fixed postoperative time point could yield misleading conclusions unless incidence is adjusted for differences in time at risk. Without accounting for differential exposure, between-group comparisons risk conflating treatment efficacy with variations in activity resumption and reinjury risk.^3^

Tracking physical activity is also valuable in older adults with conditions such as osteoarthritis (OA). While these individuals may not return to high-impact sports, changes in physical activity over time may reflect functional improvement or decline after conservative interventions such as bracing, injections, or physiotherapy^13^ or surgical interventions such as high tibial osteotomy or joint replacement. In this population, activity monitoring may provide a more complete picture of the impact of treatment beyond traditional patient-reported outcomes.

Historically, the most accurate method for measuring exposure time involved hiring field-side personnel to manually record the minutes an athlete spends at risk during practices and games.^27^ However, this method is resource-intensive, costly, and limited to supervised sport participation, often failing to capture unstructured activities that may also contribute to injury risk. Furthermore, the quality of data collected using this method may vary due to observer availability, subjective decision-making, and interrater variability.^26^ To address these limitations, accelerometer-based approaches were introduced to estimate physical activity and exposure time more objectively. Accelerometers, including wearable sensors, provide continuous movement data, offering a more comprehensive picture of an athlete's daily activity.^33^ However, these devices also have significant drawbacks, including high costs, limited battery and mechanical life, need for provision and maintenance, and practical challenges related to charging, syncing, sport-specific fitting, and participant adherence.^11,33^ These barriers restrict their accessibility and feasibility for large-scale studies or routine clinical use.

With the proliferation of smartphones equipped with built-in accelerometers, the potential for low-cost, large-scale activity tracking has increased significantly. Modern smartphones contain high-precision motion sensors, including triaxial accelerometers, gyroscopes, and a Global Positioning System, which can passively collect step count and other movement data in real time.^10,23^ Unlike dedicated accelerometers, smartphones are widely available and carried daily, reducing financial barriers for researchers and clinicians and participant burden beyond the need to download an app.^8,10,18^ These devices also enable continuous data collection, automatically logging activity without requiring manual uploads.^8,10^ Additionally, many smartphone apps integrate with other wearable devices and health databases, further expanding their utility.^10,24^

Given these advancements, the purpose of this study was to evaluate whether smartphone-based step count data could serve as a valid proxy for activity level and exposure in functionally active patients with knee OA and those recovering from ACLR. As a secondary objective, this study also aimed to evaluate patient-reported activity measures that better reflect movement type and intensity, which are not captured by step count alone. If successful, this approach could enable more accurate, scalable, and cost-effective injury surveillance and return-to-sport monitoring. We hypothesized that the app would not accurately capture all activities of high-level athletes due to limitations associated with carrying a smartphone during sport.

## Methods

### Ethics

Institutional ethics approval was obtained before study initiation (Western ReDA ID: 12560, IRB No.: R-23-175). According to the Canadian Tri-Council Policy Statement: Ethical Conduct for Research Involving Humans (TCPS 2), Article 3.3, the ability to provide consent to research is not based on age but on decision-making capacity.^16^ All patients enrolled in this study provided consent to participate without assistance.

### Participants

Patients were recruited from the clinics of 5 orthopaedic surgeons (A.G., K.R.W., R.L., J.R.G., and R.M.D.) at the Fowler Kennedy Sport Medicine Clinic in London, Ontario, Canada, from July 2023 to October 2024. Patients were eligible to participate if they were (1) within the age range of 14-44 years, 9-24 months post-isolated primary or revision ACLR, and returning to sport; or (2) at least 45 years of age, functionally active, and seeking treatment for knee OA and/or lower limb malalignment. All patients needed to be able to speak and understand English and be willing to commit to accurately recording their activities for 4 weeks. Patients were ineligible if they did not have a device that supported app downloads.

### Study Design

We conducted a prospective cohort study of functionally active patients to investigate whether a smartphone app could be used as a valid proxy for activity level and exposure time. Participants consented to track their daily activity using both the app and self-reported diaries over 4 weeks. The diary asked participants to report daily activities, for how long they participated, and whether they carried their smart device. Diaries were hosted by the data management software (EmPower Health Research, www.empowerhealthresearch.ca), and participants were registered in the database to enable direct data entry. Because it needed to serve as a reference standard and improve compliance, the database sent an automatic email reminder every 2 days, and the primary author sent a personal reminder via email if a participant missed an entry, followed by a phone call as needed. Additionally, to minimize recall bias, the database was programmed to remove participants’ access to the previous week once a new week had started.

All participants registered for the same smartphone app. Data from the app, including step count, was automatically recorded. Participants were instructed to carry their smartphones during all activities permitted by the rules of play, while diaries recorded all activities, including those in which carrying a device was not possible. Demographic and baseline clinical data, including age, sex, body mass index, and baseline activity levels, were collected to contextualize the findings.

In addition to these primary measures, the Marx Activity Rating Scale (MARS) was included as a secondary outcome to assess its ability to serve as a proxy for activity levels compared with the smartphone app. The MARS captures the frequency of specific high-intensity movements, including running, cutting, decelerating, and pivoting, over the previous 4-week period. Additionally, participants’ level of sport participation was measured using the Sport Participation questionnaire. Participants were asked to identify their sports and level of participation from the definitions in Table 1. These scales provided an additional perspective on participants’ activity intensity, offering a benchmark for evaluating the app's effectiveness in capturing diverse activity patterns. The participant completed the MARS and Sport Participation via direct entry into the electronic database.

**Table 1:** Definition of Sport Level in Sport Participation Questionnaire

Level	Definition
Elite	Highest level of professional competition (Olympics, World Games), game, practice, or training 5-7 days per week.
Varsity	Playing for a team representing a college or university, if in the United States, playing for a varsity high school team, Game, practice, or training 4-7 days per week with a coach.
Competitive	Playing for a competitive league, game, practice, or training 4-7 days per week with a coach.
Recreational	Playing for a beginner or social league, game, or practice 1-3 days per week.
Non-organized	Not playing for a league; playing casually with friends or family, game or practice <1 day per week.

### Statistical Analysis

Participants were included in the analysis if they completed at least 3 of the 4 weeks of activity tracking during which step count was also recorded. Diary-reported activity was converted into the Metabolic Equivalent of Task (MET) score.^17,21^ for each activity per minute of participation (MET-minutes) using the MET scores provided by the 2024 Compendium of Physical Activities.^17^ Patient-reported sport participation level was scored by assigning minimum and maximum scores to all sport levels. For example, participants who indicated they play sport at an elite level (game, practice, or training 5-7 days per week) were given a minimum score of 5 and a maximum score of 7 to ensure the range was covered.

Descriptive statistics were used to summarize participant characteristics and activity levels. The relationship between app-based step counts and diary-reported activity (MET-minutes) was analyzed using the Spearman correlation coefficient (ρ) and a Bland-Altman plot of *z*-scores to assess association and agreement, respectively. Similarly, correlations and plots were assessed between diary-reported activity and MARS scores, and between self-reported sport participation levels, to assess their validity as alternative proxies. The strength of associations was categorized as poor (<0.3), fair (0.3-0.5), moderate (0.6-0.8), or very strong (0.8-1.0).^6^ Given the non-normal distributions and potential nonlinear relationships among variables, all data were rank-transformed before regression analysis to allow for nonparametric modeling of monotonic associations. Linear rank regression analyses were conducted to determine whether step count data and secondary tracking measures (MARS and Sport Participation questionnaire) could predict diary-reported activity levels (MET-minutes).

#### Sample Size

The required sample size was calculated using the Bonett method for estimating agreement parameters.^5^ Using 0.85 as the benchmark level of agreement required for smartphone-derived step counts to be considered a meaningful proxy for diary-reported activity, a desired 2-sided 95% CI width of 0.15, and 2 repeated measures, the calculated sample size was 50 participants. Therefore, we aimed to recruit 100 participants total, 50 patients post-ACLR and 50 with knee OA, with additional recruitment to account for expected attrition.

## Results

A total of 116 participants were enrolled, with 62 in the OA group and 54 in the ACLR group. However, 7 OA and 7 ACLR participants did not complete the study protocol (Figure 1). Of the participants included in the analysis, 10/55 in the OA group and 0/49 in the ACLR group had bilateral affected knees. Patients were only enrolled once, even if both knees were affected. Table 2 represents the 102 participants who provided data for the study, with 55 in the OA group (44% female, mean age 57 ± 10 years) and 47 in the ACLR group (51% female, mean age 24 ± 4 years). The majority of participants in both groups identified as White (86%). Regarding daily physical demands, a greater proportion of OA participants reported moderate physical strain (36%) compared with the ACLR group (32%), while high physical strain was relatively even between groups (22% vs 21%). In terms of sport participation, the ACLR group had a higher representation of varsity (15%) and competitive-level athletes (36%). In contrast, the OA group had a greater proportion of recreational athletes (33%) or individuals who did not participate in sport (29% vs 6% in ACLR). Two participants in the OA group and 5 in the ACLR group completed only 3 out of the 4 weeks of activity tracking. For these participants, the mean of 3 weeks of data was calculated to represent their monthly tracking results.

**Figure 1.**
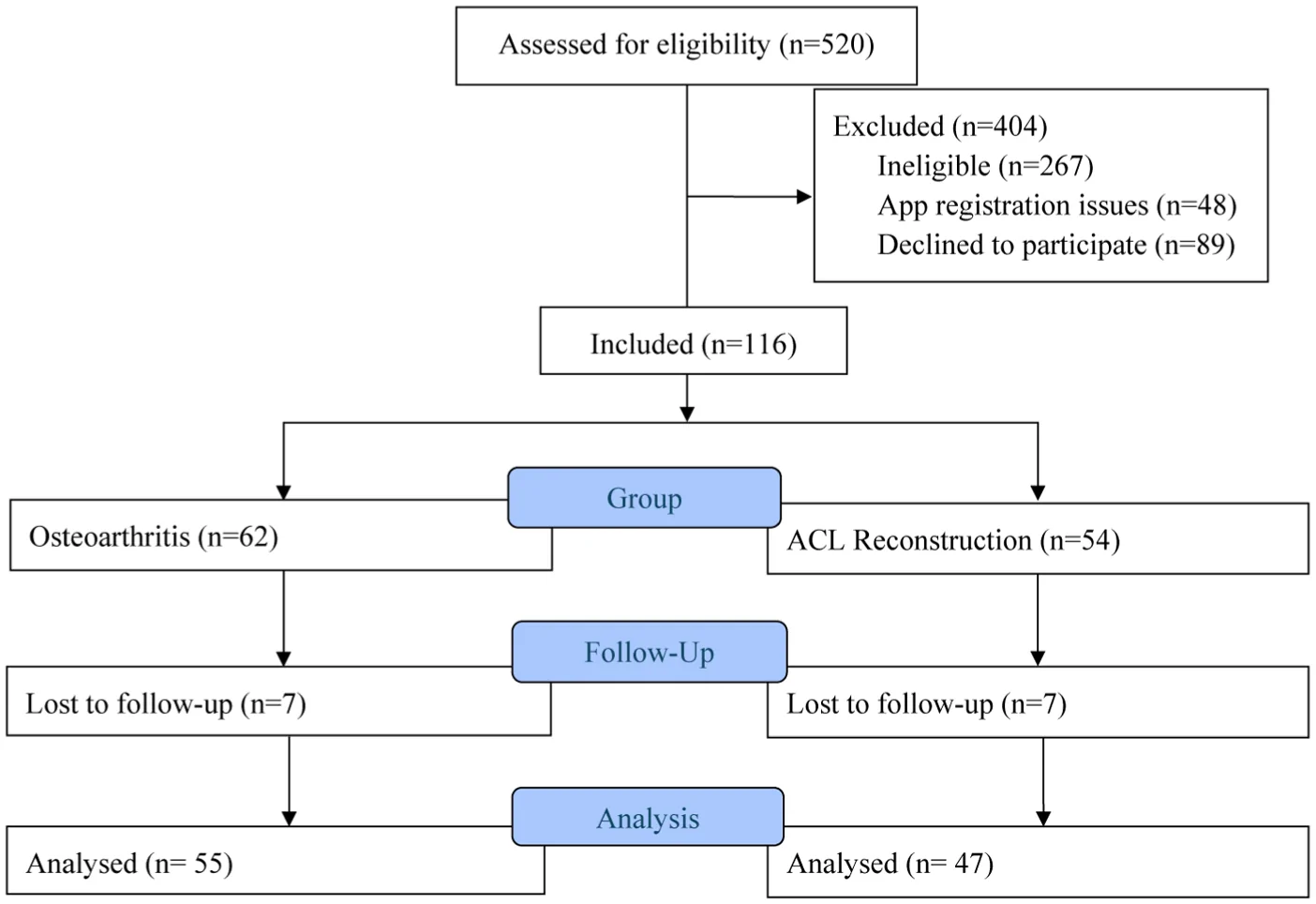
CONSORT flow diagram of patient progression through the study. CONSORT, Preferred Reporting Items for Systematic Reviews and Meta-Analyses.

**Table 2:** Participant and Baseline Characteristics for OA and ACLR Groups*^*a*^*

Baseline characteristic	OA	ACLR	Total
	n = 55	n = 47	n = 102
Sex			
Female	24 (44)	24 (51)	48 (47)
Male	31 (56)	23 (49)	54 (53)
Age, years	57.7 ± 10.8	24.2 ± 4	42.3 ± 19.5
BMI, kg/m^2^	30.5 ± 5.8	26.5 ± 5.4	28.6 ± 6.0
Race,*^*b*^*			
African American	1 (2)	2 (4)	3 (3)
Asian	0 (0)	1 (2)	1 (1)
White	49 (89)	39 (83)	88 (86)
Hispanic	1 (2)	1 (2)	2 (2)
Native American	1 (2)	0 (0)	1 (1)
Mixed	2 (4)	2 (4)	4 (4)
No response	1 (2)	2 (4)	3 (3)
Demands of daily life			
Mostly sedentary	14 (25)	16 (34)	30 (29)
Low physical strain	8 (15)	4 (9)	12 (12)
Moderate physical strain	20 (36)	15 (32)	35 (34)
High physical strain	12 (22)	10 (21)	22 (22)
No response	1 (2)	2 (4)	3 (3)
Highest sport level			
Elite	4 (7)	4 (9)	8 (8)
Varsity	2 (4)	7 (15)	9 (9)
Competitive	12 (22)	17 (36)	29 (28)
Recreational	18 (33)	12 (26)	30 (29)
Nonorganized	0 (0)	3 (6)	3 (3)
No sport	16 (29)	3 (6)	19 (19)
No response	3 (5)	1 (2)	4 (4)

a Data are presented as mean ± SD or n (%). ACLR, anterior cruciate ligament reconstruction; MARS, Marx Activity Rating Scale; OA, osteoarthritis.

b Percentages may not add up to 100% due to rounding.

The correlation between app-based step counts and diary-reported activity in MET-minutes (Figure 2) was poor and not statistically significant in both the OA group (ρ = 0.17; *P* = .20) and the ACLR group (ρ = 0.27; *P* = .07). The Bland-Altman plots (Figure 3) showed a mean difference close to zero with a narrow confidence interval about the mean difference for both the OA group (d̄ = −0.10 [95% CI, −0.43 to 0.23]) and the ACLR group (d̄ = −0.17 [95% CI, 0.14 to 0.49]), indicating very minimal systematic bias and strong agreement between activity tracking methods on a group level. However, the limits of agreement (LoA) were wide for both groups, ranging from −2.49 to 2.29 in the OA group and −1.93 to 2.28 in the ACLR group. This indicates that individual-level differences between app- and diary-based activity estimates can exceed ±2 standard deviations, limiting their interchangeability for patient-specific monitoring.

**Figure 2.**
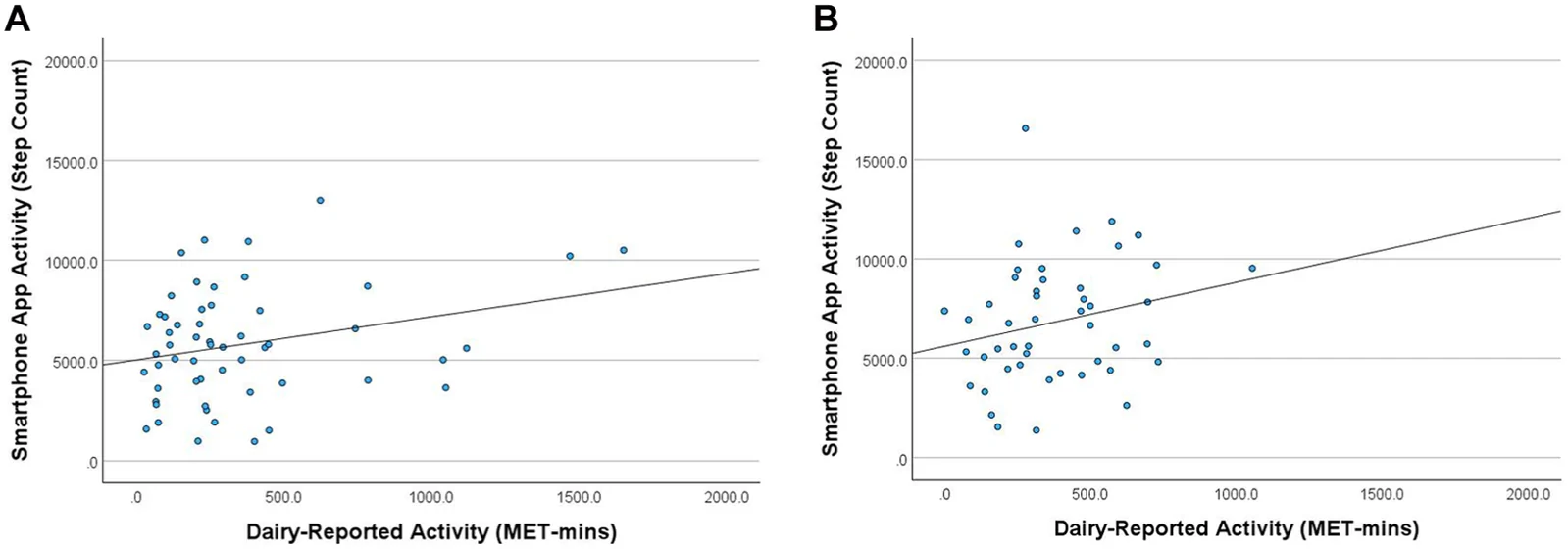
(A) A scatterplot displaying the correlation between diary-reported activity and app-reported step count for the osteoarthritis group. (B) A scatterplot displaying the correlation between diary-reported activity and app-reported step count for the anterior cruciate ligament reconstruction group. ACLR, anterior cruciate ligament reconstruction; MET, metabolic equivalent of task.

**Figure 3.**
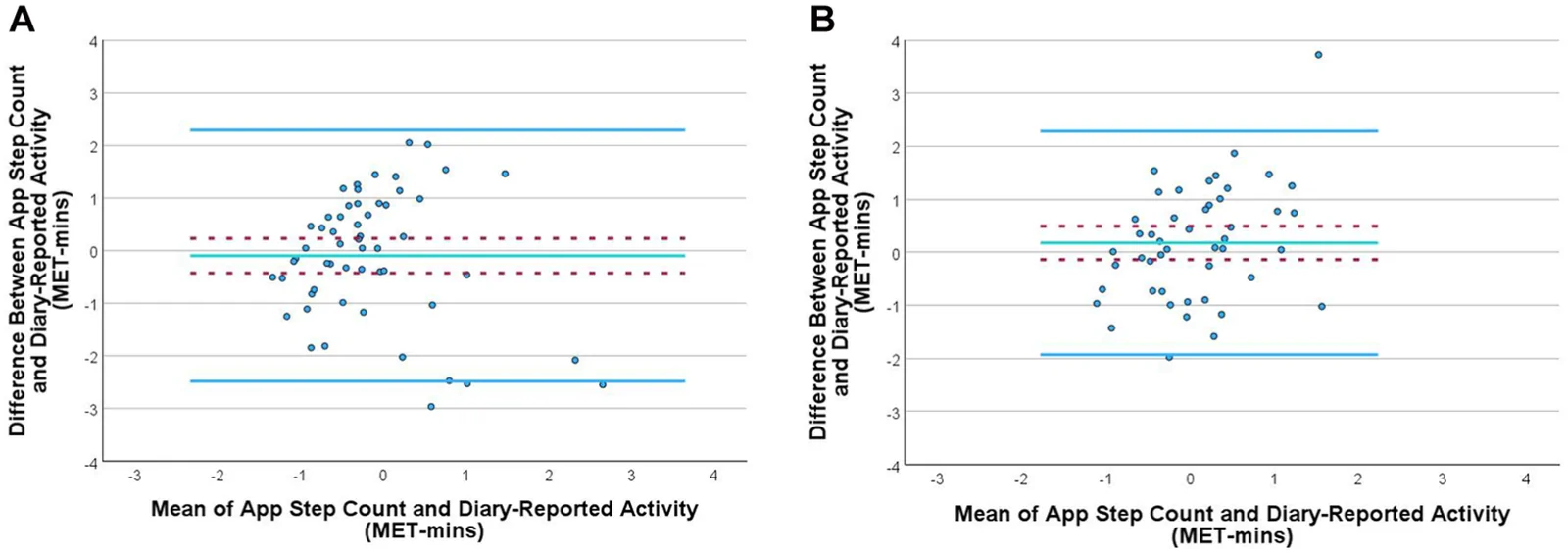
(A) Bland-Altman plots of agreement between standardized diary-reported activity and app-reported step count (*Z*-score) for the osteoarthritis group, with a solid turquoise line representing the mean difference, a dashed red line representing the 95% CI about the mean difference, and the solid blue lines representing the upper and lower limits of agreement. (B) Bland-Altman plots of agreement between standardized diary-reported activity and app-reported step count (*Z*-score) for the ACLR group, with a solid turquoise line representing the mean difference, a dashed red line representing the 95% CI about the mean difference, and the solid blue lines representing the upper and lower limits of agreement. ACLR, anterior cruciate ligament reconstruction.

The magnitude of the association between MARS scores and diary-reported activity (Figure 4) was moderate and statistically significant in both the OA (ρ = 0.33; *P* = .02) and the ACLR groups (ρ = 0.45; *P* = .002), with a stronger correlation in the ACLR group. Additionally, the Bland-Altman plots comparing the MARS score to the MET-minutes from the activity diaries (Figure 5) show a mean difference close to zero with a narrow confidence interval about the mean difference for both the OA (d̄ = 0.09 [95% CI, −0.28 to 0.46]) and the ACLR group (d̄ = −0.03 [95% CI, −0.31 to 0.25]), indicating minimal systematic bias and strong agreement between activity tracking methods on a group level. However, as with the app-reported step count, the LoA are wide for both groups, ranging from −2.61 to 2.79 in the OA group and −1.87 to 1.82 in the ACLR group, suggesting considerable individual-level variability and limiting the interchangeability of MARS and MET-minutes for patient-specific monitoring. Additionally, the fanning pattern in the left plot indicates less agreement at higher activity levels in the OA group when comparing MARS to diary-reported MET-minutes. It suggests the MARS score might under- or overestimate actual activity depending on the intensity level.

**Figure 4.**
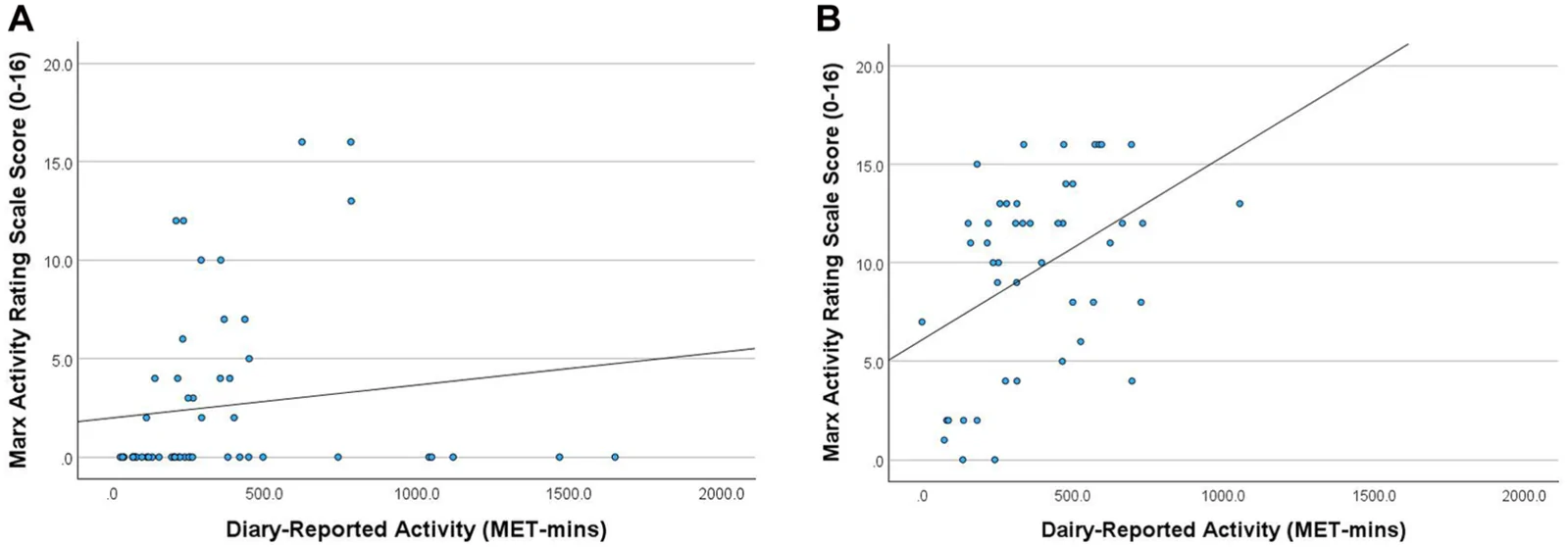
(A) Scatterplot displaying the correlation between diary-reported activity (MET-minutes) and MARS Score (0-16) for the OA group. (B) Scatterplot displaying the correlation between diary-reported activity (MET-minutes) and MARS Score (0-16) for the ACLR group. ACLR, anterior cruciate ligament reconstruction; MARS score, Marx Activity Rating Scale score; MET, metabolic equivalent of task; OA, osteoarthritis.

**Figure 5.**
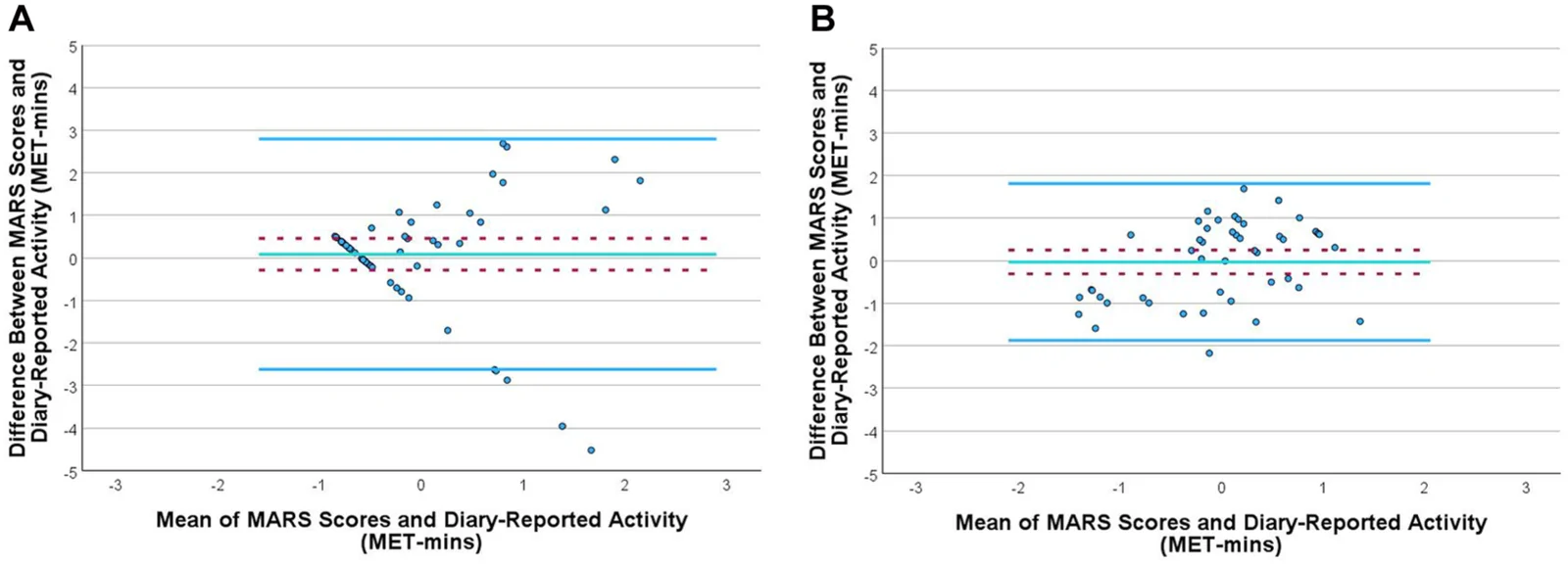
(A) Bland-Altman plots of agreement between standardized diary-reported activity and MARS score (*Z*-score) for the OA group, with a solid turquoise line representing the mean difference, a dashed red line representing the 95% CI about the mean difference, and the solid blue lines representing the upper and lower limits of agreement. (B) Bland-Altman plots of agreement between standardized diary-reported activity and MARS score (*Z*-score) for the ACLR group, with a solid turquoise line representing the mean difference, a dashed red line representing the 95% CI about the mean difference, and the solid blue lines representing the upper and lower limits of agreement. ACLR, anterior cruciate ligament reconstruction; MARS score, Marx Activity Rating Scale score; MET, metabolic equivalent of task; OA, osteoarthritis.

The magnitude of the association between the minimum (min) and maximum (max) sport participation and diary-reported activity found the correlation was poor and not statistically significant in the OA group (min: ρ = 0.17; *P* = .22, max: ρ = 0.16; *P* = .25) but moderate and statistically significant in the ACLR group (min: ρ = 0.39; *P* = .006, max: ρ = 0.39; *P* = .007) (Figures 6). The Bland-Altman plots showed mean differences near zero and narrow 95% CIs, indicating minimal systematic bias and strong group-level agreement (Figure 7). In the OA group, the mean difference was 0.09 for both the highest (95% CI, −0.32 to 0.50; LoA: 95% CI, −2.88 to 3.06) and lowest (95% CI, −0.32 to 0.50; LoA: −2.84 to 3.02) reported sport participation levels. In the ACLR group, the mean difference was 0.03 for the highest ([95% CI, −0.28 to 0.34]; LoA [–2.02 to 2.09]) sport participation levels and 0.04 for the lowest ([95% CI, −0.27 to 0.34]; LoA [–1.99 to 2.06]) sport participation levels. However, visual inspection of the Bland-Altman plots revealed a fanning pattern in the OA group, suggesting potential heteroscedasticity, with agreement between methods decreasing at higher activity levels. Additionally, all subgroups exhibited wide limits of agreement, suggesting that individual-level discrepancies between activity measures can exceed ±2 units, again limiting their interchangeability for patient-specific tracking.

**Figure 6.**
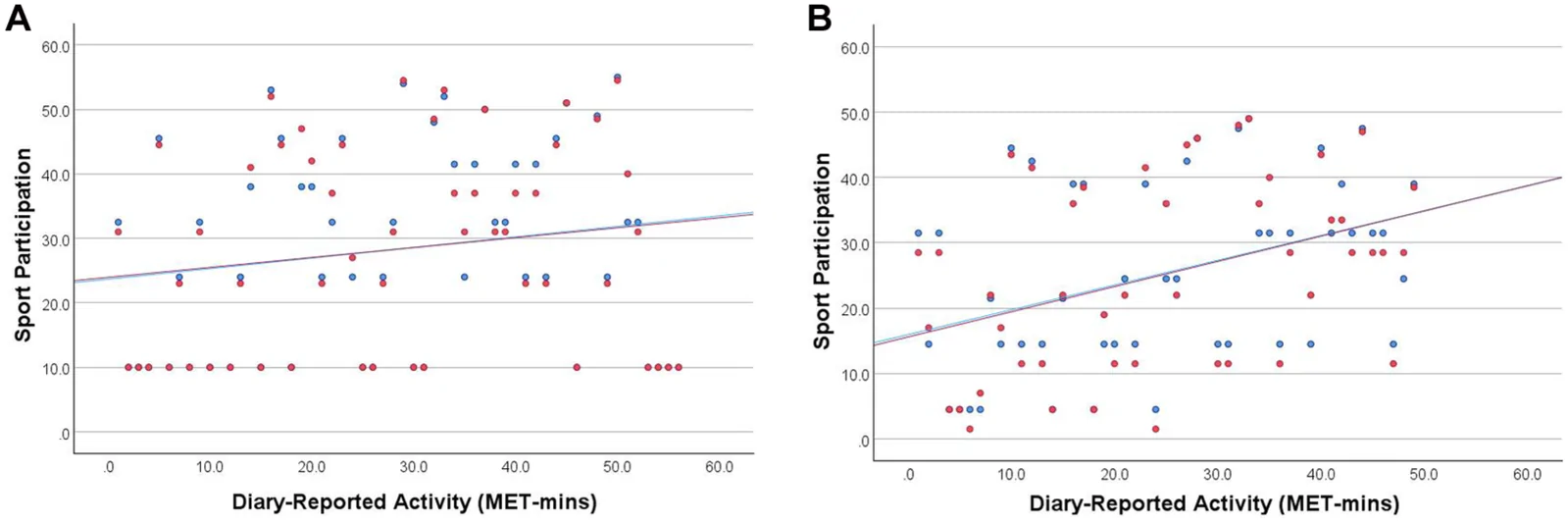
(A) Scatterplot displaying the correlation between diary-reported activity (MET-minutes) and the highest-reported sport participation (red) and lowest-reported sport participation (blue) for the OA group. (B) Scatterplot displaying the correlation between diary-reported activity (MET-minutes) and the highest-reported sport participation (red) and lowest-reported sport participation (blue) for the ACLR group. ACLR, anterior cruciate ligament reconstruction; MET, metabolic equivalent of task; OA, osteoarthritis.

**Figure 7.**
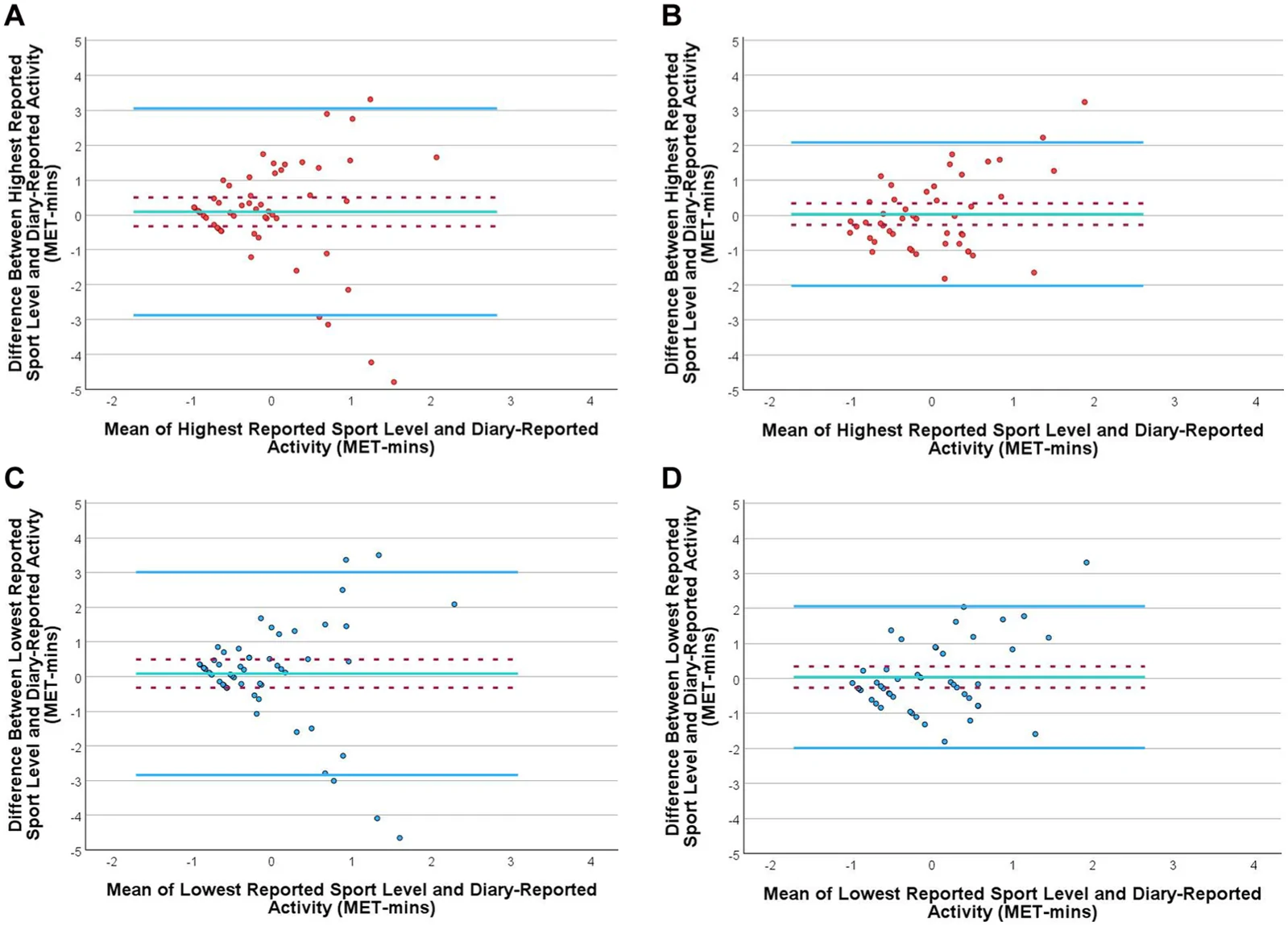
(A) Bland-Altman plots of agreement between standardized diary-reported activity and highest reported sport participation (*Z*-score) for the OA group, with a solid turquoise line representing the mean difference, dashed red line representing the 95% CI about the mean difference, and the solid blue lines representing the upper and lower limits of agreement. (B) Bland-Altman plots of agreement between standardized diary-reported activity and highest reported sport participation (*Z*-score) for the ACLR group, with a solid turquoise line representing the mean difference, a dashed red line representing the 95% CI about the mean difference, and the solid blue lines representing the upper and lower limits of agreement. (C) Bland-Altman plots of agreement between standardized diary-reported activity and lowest reported sport participation (*Z*-score) for the OA group, with a solid turquoise line representing the mean difference, a dashed red line representing the 95% CI about the mean difference, and the solid blue lines representing the upper and lower limits of agreement. (D) Bland-Altman plots of agreement between standardized diary-reported activity and lowest reported sport participation (Z-score) for the ACLR group, with a solid turquoise line representing the mean difference, a dashed red line representing the 95% CI about the mean difference, and the solid blue lines representing the upper and lower limits of agreement. ACLR, anterior cruciate ligament reconstruction; OA, osteoarthritis.

Rank-based regression analyses were performed to examine whether the combination of app-collected step data and secondary tracking measures, MARS scores, and self-reported sport participation levels could predict diary-reported MET-minutes. In the univariate models, MARS scores were statistically significant in both the OA (β = 0.38 [95% CI, 0.07 to 0.69]; *P* = .02) and ACLR (β = 0.45 [95% CI, 0.20 to 0.79]; *P* = .002) groups. Minimum and maximum self-reported sport participation was not significantly associated with MET-minutes in the OA group (min: β = 0.18 [95% CI, −0.12 to 0.46]; *P* = .22, max: β = 0.17 [95% CI, −0.12 to 0.45]; *P* = .25), but was in the ACL group (min: β = 0.4 [95% CI, 0.12 to 0.69]; *P* = .007, max: β = 0.38 [95% CI, 0.11 to 0.66]; *P* = .007), but not in the OA group. Step count data were not significantly associated with MET-minutes in univariate analysis for the OA group (β = 0.18 [95% CI, −0.10 to 0.45]; *P* = .20) or the ACL group (β = 0.27 [95% CI, −0.03 to 0.56]; *P* = .07). When included together in a multivariable model, MARS scores (Table 3) retained significance for both groups and sport participation (Table 4) retained significance for the ACL group only, while step count remained nonsignificant.

**Table 3:** Multivariate Rank Regression Analysis of App-Collected Step Counts and MARS Scores Associated With Diary-Reported Activity (MET-minutes) in the OA Group and the ACL Group^a^

Variable	Coefficient	95% CI	*P*
OA Group			
Smartphone step count	0.20	−0.07 to 0.47	.14
MARS score	0.39	0.09 to 0.70	.01
ACLR Group			
Smartphone step count	0.20	−0.08 to 0.47	.47
MARS score	0.46	0.16 to 0.76	.003

a ACLR, anterior cruciate ligament reconstruction; MARS, Marx Activity Rating Scale; OA, osteoarthritis.

**Table 4:** Multivariate Rank Regression Analysis of App-Collected Step Counts and Sport Participation Scores Associated With Diary-Reported Activity (MET-minutes) in the OA Group (4A, 4B) and the ACLR Group (4C, 4D)^a^

4A. Variable	Coefficient	95% CI	*P*
Smartphone step count	0.19	−0.09 to 0.46	.18
Highest sport level	0.37	−0.13 to 0.44	.28
4B. Variable	Coefficient	95% CI	*P*
Smartphone step count	0.18	−0.09 to 0.46	.19
Lowest sport level	0.16	−0.12 to 0.45	.26
4C. Variable	Coefficient	95% CI	*P*
Smartphone step count	0.24	−0.03 to 0.52	.08
Highest sport level	0.37	0.10 to 0.64	.008
4D. Variable	Coefficient	95% CI	*P*
Smartphone step count	0.23	−0.04 to 0.51	.09
Lowest sport level	0.39	0.10 to 0.66	.009

a The first table in each group presents the highest reported sport level, followed by the lowest. ACLR, anterior cruciate ligament reconstruction; OA, osteoarthritis.

### Discussion

The major findings of our study demonstrated that, compared with detailed activity diaries, smartphone-recorded step counts showed a nonsignificant, poor correlation in the OA and ACLR groups, respectively, and wide individual-level variability, limiting their clinical utility for precise monitoring. Additionally, app-collected step count was not predictive of diary-reported activity when analyzed alone or when combined with additional activity measures such as the self-reported MARS or sport participation questionnaires. However, there was minimal systematic bias and strong group-level agreement between the app-step data and diary-reported activity, suggesting that smartphone-based step counting may still be valuable for population-level surveillance or large-scale trials. Additionally, the MARS showed a moderate, statistically significant correlation and predictive ability with diary-derived MET-minutes, especially in the ACLR group. This suggests that movement-specific patient-reported outcome measures (PROMs) may better capture the intensity of activity in athletic populations. Perceived sport participation levels were moderately correlated and predictive in the ACLR group but poorly correlated and not predictive in the OA group.

Our findings are consistent with previous research highlighting the limitations of smartphone-based step counting in capturing complex, sport-specific activity. In a recent systematic review, Prado et al^31^ found that while smartphone accelerometers demonstrate acceptable validity for walking and basic gait analysis, their accuracy significantly decreases during vigorous activities, especially when the device is carried inconsistently or not worn at all. Similarly, Donaire-Gonzalez et al^12^ showed that although smartphones provide accurate data for low- to moderate-intensity walking, they lack the fidelity needed to quantify more dynamic movements and often underestimate true physical activity when movement intensity increases or when the phone is not worn on the body during the task. These observations are consistent with our participants who engaged in formal sports, where phone carriage was infeasible, and likely explain the poor associations found between step count and activity diaries in our cohort. We expected that higher-level athletes would record more steps overall, even if their phones did not capture activity during formal sport. However, this was not supported.

Colley et al^7^ noted that studies using accelerometers often involve small sample sizes due to high device costs and logistical constraints, which limit generalizability and feasibility for large-scale surveillance. The minimal systematic bias observed in both patient populations on the Bland-Altman plots suggests that while individual estimates may be imprecise, group-level agreement is acceptable. This supports potential use in research settings where population-level trends are of greater interest than individual outcomes. Importantly, this has implications for scalability. Additionally, the app leverages participants’ smartphones to passively and continuously collect activity data, reducing costs and participant burden. Therefore, the app may represent a pragmatic, low-cost solution for measuring physical activity and exposure time in large epidemiologic studies, clinical registries, and pragmatic trials where precise individual measurement is not required, and reliable group-level estimates are sufficient.

The MARS was designed to capture high-level activity patterns in orthopaedic populations using brief, patient-reported metrics and has demonstrated strong group-level validity for clinical decision-making and comparisons across cohorts.^25^ This aligns with our finding of a strong, significant correlation between MARS scores and patient-reported activity diaries, particularly in the ACLR group, where the movements assessed by the MARS reflect the high-risk activities common in the sports to which participants were likely returning and in which they were unable to carry their phones.

This study has several strengths. We collected data prospectively and included 2 clinically distinct populations: young patients recovering from ACLR and older adults with knee OA, allowing for evaluation across a broad spectrum of functional levels and activity types. Additionally, using multiple complementary methods to assess exposure enabled a thorough evaluation of validity and agreement among commonly used tools.

There were also several limitations to our study. Although the attrition rate was low (14%), the small sample in each group could influence the precision of the analysis. A total of 48 people were excluded from the study due to app registration issues, potentially introducing selection bias. Additionally, data completeness from the step count app was affected by a platform-related issue that caused participants to be unexpectedly logged out during the tracking period. These technical limitations resulted in loss of app-based data for some participants, which may have influenced the correlation and agreement with diary-reported activity. The location (pocket, hand, bag, etc.) and operating system (Android, iOS, etc.) of smartphones were not standardized to prioritize pragmatism, which may have introduced variability in the devices’ ability to accurately track activity. However, previous validation research found that neither smartphone position nor operating system affected step-detection accuracy.^1,19^ While the app provided passive, objective data, it did not capture activity context or intensity, limiting its ability to reflect nonambulatory or sport-specific exposure. This could contribute to a “floor effect” for those participating in high-effort but low-step activities (eg, swimming, cycling). These factors should be considered when interpreting the performance of app-based tracking in functionally active populations. In addition, studies have shown that patient-reported diaries can lack validity, particularly when compliance is poor or recall is inaccurate.^28^ With this knowledge, the study team took several steps to overcome these shortcomings. First, as part of the consent process, the research team explained the study hypothesis and the importance of accurately recounting daily activities. Second, electronic diaries were used because they had a higher compliance rate than paper diaries.^32^ Third, the participants received automatic e-mail reminders to complete their diaries. Finally, the primary author conducted daily monitoring of diary completion and contacted participants by telephone if any diaries were incomplete. Despite these extraordinary measures, recall or social desirability bias may have been present.

Future research should evaluate group-level validity in broader, more diverse cohorts and explore whether combining step counts with other smartphone or wearable sensor data improves activity capture. Longitudinal studies are needed to determine whether changes in app-based metrics reflect true functional improvements over time, especially in the context of rehabilitation or return to sport. Additionally, exploring barriers to phone carry and compliance, particularly during high-risk or sport-specific activities, could inform strategies to improve data completeness, such as passive-tracking enhancements or self-report prompts.

## Conclusion

Our study demonstrated acceptable group-level agreement between the smartphone app and diary-based activity but insufficient precision for individual patient monitoring. MARS outperformed an app-based step count as a proxy for exposure time, particularly in athletes. Therefore, smartphone-based tracking is a pragmatic, low-cost option for large-scale trials and epidemiologic surveillance focused on group-level trends, whereas movement-specific PROMs (eg, MARS) may better capture exposure to individual activity levels in athletic populations.
